# Supplementary material for: Stomatal responses of differently CO2-acclimated plants to natural and experimental CO2 gradients
Source: PLoS One. 2026 Apr 22;21(4):e0346112. doi: 10.1371/journal.pone.0346112 (PMC13102186; doi:10.1371/journal.pone.0346112)
Supplement: S1 Fig — The altitudinal distribution of the examined taxa is based on records from the National Data and Information Center on the Swiss Flora (www.infoflora.ch). Anthyllis vulneraria subsp. carpatica (A. carpatica): n = 3,044 observations; Anthyllis vulneraria subsp. valesiaca (A. valesiaca): n = 1,366 observations; Arabidopsis thaliana (A. thaliana): n = 883 observations; Arabis alpina (A. alpina): n = 1,078 observations. (PDF) [file pone.0346112.s001.pdf]

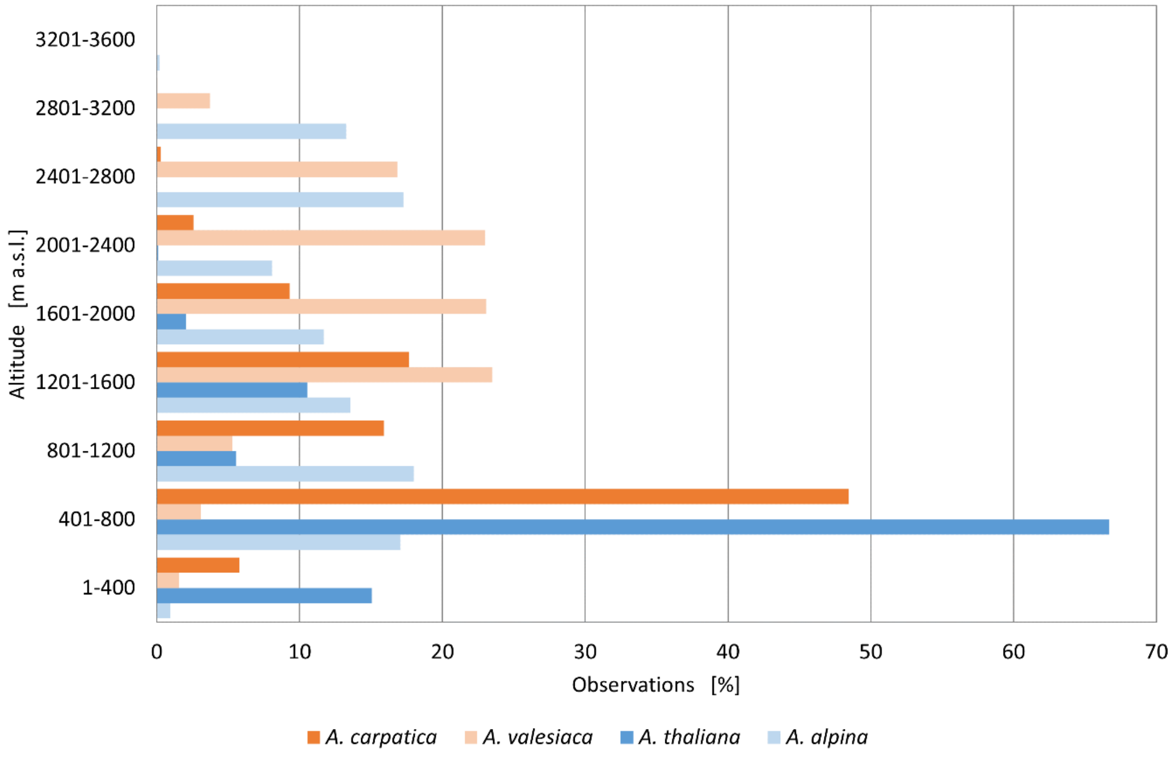

S1 Fig. Altitudinal distribution of the taxa under study. The altitudinal distribution of the examined taxa is based on records from the National Data and Information Center on the Swiss Flora (www.infoflora.ch).

Anthyllis vulneraria subsp. *carpatica* (A. carpatica): *n* = 3,044 observations; *Anthyllis vulneraria* subsp. *valesiaca* (A. valesiaca): *n* = 1,366 observations; *Arabidopsis thaliana* (A. thaliana): *n* = 883 observations; *Arabis alpina* (A. alpina): *n* = 1,078 observations
