# Supplementary material for: Stomatal responses of differently CO2-acclimated plants to natural and experimental CO2 gradients
Source: PLoS One. 2026 Apr 22;21(4):e0346112. doi: 10.1371/journal.pone.0346112 (PMC13102186; doi:10.1371/journal.pone.0346112)
Supplement: S2 Fig — Epidermal cell density (n = 828) was measured in high-altitude taxa (Anthyllis vulneraria subsp. valesiaca and Arabis alpina, altitude of origin 2,970 m a.s.l.) and low-altitude taxa (Anthyllis vulneraria subsp. carpatica, 540 m a.s.l., and Arabidopsis thaliana Col-0). Plants were cultivated under reduced (30 Pa) and ambient pCO₂ (42 Pa). Marginal means along with their 95% confidence intervals are presented. Statistical analysis results are provided in S7 Table. (PDF) [file pone.0346112.s002.pdf]

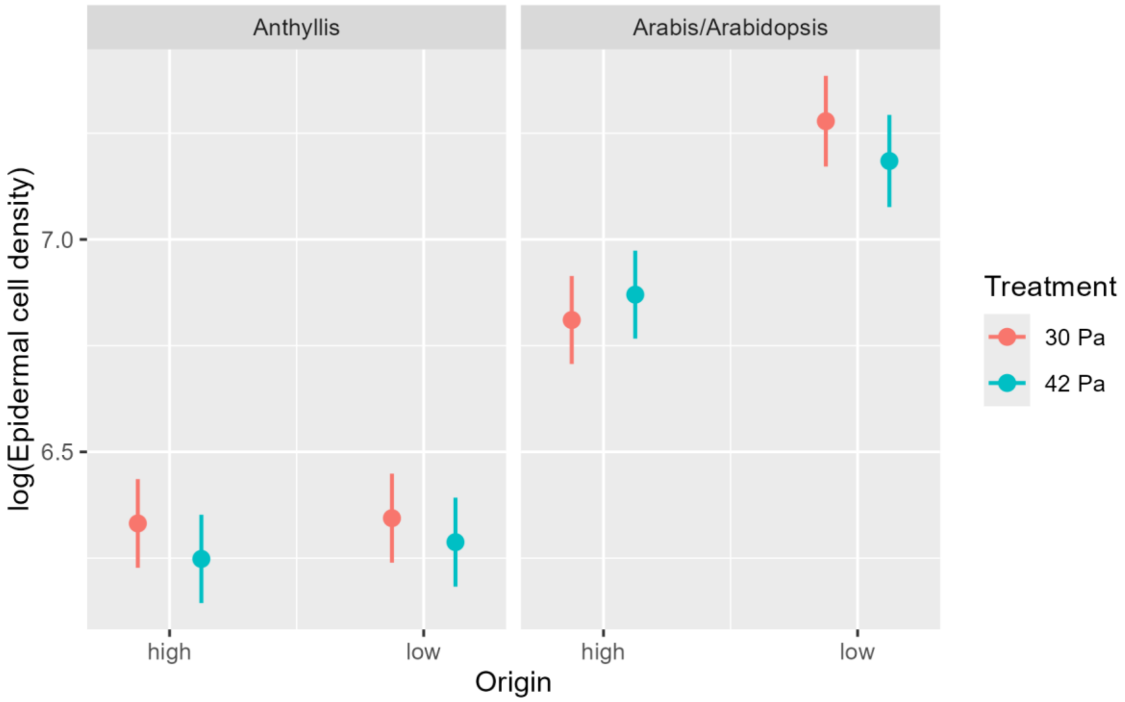

S2 Fig. Response of epidermal cell density to variations in pCO_2. Epidermal cell density (n = 828) was measured in high-altitude taxa (*Anthyllis vulneraria* subsp. *valesiaca* and *Arabis alpina*, altitude of origin 2,970 m a.s.l.) and low-altitude taxa (*Anthyllis vulneraria* subsp. *carpatica*, 540 m a.s.l., and *Arabidopsis thaliana* Col-0). Plants were cultivated under reduced (30 Pa) and ambient pCO_2 (42 Pa). Marginal means along with their 95% confidence intervals are presented. Statistical analysis results are provided in S7 Table.
