# Supplementary material for: Stomatal responses of differently CO2-acclimated plants to natural and experimental CO2 gradients
Source: PLoS One. 2026 Apr 22;21(4):e0346112. doi: 10.1371/journal.pone.0346112 (PMC13102186; doi:10.1371/journal.pone.0346112)
Supplement: S1 Table — All collection sites are located in Switzerland (BE: Canton of Berne; VS: Canton of Valais). The areas entered for the sampling of leaves and seeds are not privately owned and are freely accessible. The taxa sampled are not protected by law. (PDF) [file pone.0346112.s003.pdf]

S1 Table. Sites of collection of seeds and leaves for stomatal frequency analysis.

Taxon	Site and altitude	Habitat type
Anthyllis vulneraria subsp. carpatica (Pant.) Nyman	Bremgarten BE, Hoger<br>540 m a. s. l.	mesophile hay meadow
Anthyllis vulneraria subsp. valesiaca (Beck) Guyot	Zermatt VS, Gornerli<br>2,970 m a. s. l.	open alpine grassland on acid soil
Arabidopsis thaliana (L.) Heynh.	Rubigen BE, Thunstrasse<br>540 m a. s. l.	road embankment with open sward
Arabis alpina L.	Lauterbrunnen BE, Schilthorn<br>2,970 m a. s. l.	calcareous scree community

All collection sites are located in Switzerland (BE: Canton of Berne; VS: Canton of Valais). The areas entered for the sampling of leaves and seeds are not privately owned and are freely accessible. The taxa sampled are not protected by law.
