# Supplementary material for: Stomatal responses of differently CO2-acclimated plants to natural and experimental CO2 gradients
Source: PLoS One. 2026 Apr 22;21(4):e0346112. doi: 10.1371/journal.pone.0346112 (PMC13102186; doi:10.1371/journal.pone.0346112)
Supplement: S2 Table — (PDF) [file pone.0346112.s004.pdf]

S2 Table. Composition of the nutrient solution used in hydroponic plant cultivation.

Concentration	Nutrient
1.5 mM	$Ca(NO_3)_2\ 4H_2O$
1 mM	KNO_3
0.75 mM	KH_2PO_4
0.75 mM	$MgSO_4\ 7H_2O$
30 µM	Fe^{3+} ethylenediaminetetraacetic acid
2 µM	H_3BO_3
0.4 µM	$MnCl_2\ 4H_2O$
0.08 µM	MoO_3
0.07 µM	$ZnSO_4\ 7H_2O$
0.05 µM	$CuSO_4\ 5H_2O$
