# Supplementary material for: Stomatal responses of differently CO2-acclimated plants to natural and experimental CO2 gradients
Source: PLoS One. 2026 Apr 22;21(4):e0346112. doi: 10.1371/journal.pone.0346112 (PMC13102186; doi:10.1371/journal.pone.0346112)
Supplement: S3 Table — Type-I ANOVA of the linear mixed model testing the impact of Origin, Taxon and pCO2 treatment on stomatal aperture (SA). (PDF) [file pone.0346112.s005.pdf]

S3 Table. Stomatal aperture response to variations in pCO_2.

**Stomatal aperture (SA); n = 800**				
	numDF	denDF	F-value	p-value
Intercept	1	791	5114.442	<.0001
Origin	1	791	100.933	<.0001
Taxon	1	791	33.500	<.0001
Treatment (pCO_2)	1	791	1.776	0.1830
Origin x Taxon	1	791	8.751	0.0032
Origin × Treatment (pCO_2)	1	791	1.132	0.2877
Taxon × Treatment (pCO_2)	1	791	7.691	0.0057
Origin x Taxon × Treatment (pCO_2)	1	791	2.581	0.1086

Type-I ANOVA of the linear mixed model testing the impact of Origin, Taxon and pCO_2 treatment on stomatal aperture (SA).
