# Supplementary material for: Stomatal responses of differently CO2-acclimated plants to natural and experimental CO2 gradients
Source: PLoS One. 2026 Apr 22;21(4):e0346112. doi: 10.1371/journal.pone.0346112 (PMC13102186; doi:10.1371/journal.pone.0346112)
Supplement: S4 Table — Type-I ANOVA of the linear model testing the impact of Origin and Taxon on stomatal density (SD; log-transformed) and stomatal index (SI) of plants growing at their natural sites. (PDF) [file pone.0346112.s006.pdf]

S4 Table. Stomatal frequency of plants growing at their natural sites.

**Stomatal density (SD); n = 82**					
	Df	Sum Sq	Mean Sq	F value	Pr(>F)
Origin	1	0.5138	0.5138	5.6055	0.02038
Taxon	1	9.7182	9.7182	106.0349	3.395e-16
Origin × Taxon	1	0.5347	0.5347	5.8345	0.01805

**Stomatal index (SI); n = 82**					
	Df	Sum Sq	Mean Sq	F value	Pr(>F)
Origin	1	0.018350	0.018350	19.209	3.614e-05
Taxon	1	0.072189	0.072189	75.567	4.257e-13
Origin × Taxon	1	0.029103	0.029103	30.464	4.293e-07

Type-I ANOVA of the linear model testing the impact of Origin and Taxon on stomatal density (SD; log-transformed) and stomatal index (SI) of plants growing at their natural sites.
