# Supplementary material for: Stomatal responses of differently CO2-acclimated plants to natural and experimental CO2 gradients
Source: PLoS One. 2026 Apr 22;21(4):e0346112. doi: 10.1371/journal.pone.0346112 (PMC13102186; doi:10.1371/journal.pone.0346112)
Supplement: S5 Table — Type-I ANOVA of the linear model testing for differences in stomatal density (SD; log-transformed) and stomatal index (SI) between natural populations and their corresponding pCO2 treatment in the growth chamber (Treatment) for each Taxon. A CO2 partial pressure of 30 Pa corresponds to the high-altitude population while 42 Pa corresponds to the low-altitude population. (PDF) [file pone.0346112.s007.pdf]

**S5 Table. Stomatal frequency of plants from natural sites and their laboratory-grown conspecifics exposed to altitude-specific $pCO_2$ levels.**

| **A Stomatal density (SD), 30 Pa / nat. site; n = 262** | | | | | |
|---|---|---|---|---|---|
| | Df | Sum Sq | Mean Sq | F value | Pr(>F) |
| Taxon | 1 | 18.92 | 18.9199 | 86.2332 | <2e-16 |
| Treatment ($pCO_2$) | 1 | 0.108 | 0.1079 | 0.4919 | 0.4837 |
| Taxon × Treatment ($pCO_2$) | 1 | 0.001 | 0.0006 | 0.0025 | 0.9601 |

| **B Stomatal index (SI), 30 Pa / nat. site; n = 254** | | | | | |
|---|---|---|---|---|---|
| | Df | Sum Sq | Mean Sq | F value | Pr(>F) |
| Taxon | 1 | 0.044624 | 0.044624 | 56.6810 | 9.396e-13 |
| Treatment ($pCO_2$) | 1 | 0.000990 | 0.000990 | 1.2579 | 0.2631 |
| Taxon × Treatment ($pCO_2$) | 1 | 0.000228 | 0.000228 | 0.2895 | 0.5910 |

| **C Stomatal density (SD), 42 Pa / nat. site; n = 239** | | | | | |
|---|---|---|---|---|---|
| | Df | Sum Sq | Mean Sq | F value | Pr(>F) |
| Taxon | 1 | 60.256 | 60.256 | 297.3518 | <2e-16 |
| Treatment ($pCO_2$) | 1 | 0.731 | 0.731 | 3.6080 | 0.05873 |
| Taxon × Treatment ($pCO_2$) | 1 | 0.275 | 0.275 | 1.3570 | 0.24525 |

| **D Stomatal index (SI), 42 Pa / nat. site; n = 239** | | | | | |
|---|---|---|---|---|---|
| | Df | Sum Sq | Mean Sq | F value | Pr(>F) |
| Taxon | 1 | 0.101669 | 0.101669 | 145.5121 | <2.2e-16 |
| Treatment ($pCO_2$) | 1 | 0.013808 | 0.013808 | 19.7618 | 1.355e-05 |
| Taxon × Treatment ($pCO_2$) | 1 | 0.038567 | 0.038567 | 55.1991 | 2.029e-12 |

Type-I ANOVA of the linear model testing for differences in stomatal density (SD; log-transformed) and stomatal index (SI) between natural populations and their corresponding $pCO_2$ treatment in the growth chamber (Treatment) for each Taxon. A $CO_2$ partial pressure of 30 Pa corresponds to the high-altitude population while 42 Pa corresponds to the low-altitude population.
