# Supplementary material for: Stomatal responses of differently CO2-acclimated plants to natural and experimental CO2 gradients
Source: PLoS One. 2026 Apr 22;21(4):e0346112. doi: 10.1371/journal.pone.0346112 (PMC13102186; doi:10.1371/journal.pone.0346112)
Supplement: S6 Table — Type-I ANOVA of the linear mixed model testing the impact of Origin, Taxon and pCO2 treatment on stomatal density (SD; log-transformed) and stomatal index (SI). (PDF) [file pone.0346112.s008.pdf]

S6 Table. Stomatal frequency response to variations in pCO_2.

**Stomatal density (SD); n = 836**				
	numDF	denDF	F-value	p-value
Intercept	1	787	69149.03	<.0001
Origin	1	787	30.03	<.0001
Taxon	1	787	516.98	<.0001
Treatment (pCO_2)	1	787	22.35	<.0001
Origin × Taxon	1	39	41.93	<.0001
Origin × Treatment (pCO_2)	1	787	0.97	0.3257
Taxon × Treatment (pCO_2)	1	787	0.19	0.6652
Origin × Taxon × Treatment (pCO_2)	1	787	4.38	0.0368

**Stomatal index (SI); n = 828**				
	numDF	denDF	F-value	p-value
Intercept	1	779	2871.6120	<.0001
Origin	1	779	0.6522	0.4196
Taxon	1	779	213.4387	<.0001
Treatment (pCO_2)	1	779	86.4159	<.0001
Origin × Taxon	1	39	11.9895	0.0013
Origin × Treatment (pCO_2)	1	779	0.0295	0.8638
Taxon × Treatment (pCO_2)	1	779	9.4369	0.0022
Origin × Taxon × Treatment (pCO_2)	1	779	2.4124	0.1208

Type-I ANOVA of the linear mixed model testing the impact of Origin, Taxon and pCO_2 treatment on stomatal density (SD; log-transformed) and stomatal index (SI).
