# Supplementary material for: Stomatal responses of differently CO2-acclimated plants to natural and experimental CO2 gradients
Source: PLoS One. 2026 Apr 22;21(4):e0346112. doi: 10.1371/journal.pone.0346112 (PMC13102186; doi:10.1371/journal.pone.0346112)
Supplement: S7 Table — Type-I ANOVA of the linear mixed model testing the impact of Origin, Taxon and pCO2 treatment on epidermal cell density (log-transformed). (PDF) [file pone.0346112.s009.pdf]

**S7 Table. Response of epidermal cell density to variations in $pCO_2$.**

| **Epidermal cell density; n = 828** | | | | |
|---|---|---|---|---|
| | numDF | denDF | F-value | p-value |
| Intercept | 1 | 779 | 39414.70 | <.0001 |
| Origin | 1 | 779 | 31.47 | <.0001 |
| Taxon | 1 | 779 | 476.44 | <.0001 |
| Treatment ($pCO_2$) | 1 | 779 | 2.02 | 0.1558 |
| Origin x Taxon | 1 | 39 | 30.25 | <.0001 |
| Origin × Treatment ($pCO_2$) | 1 | 779 | 1.11 | 0.2914 |
| Taxon × Treatment ($pCO_2$) | 1 | 779 | 0.87 | 0.3520 |
| Origin x Taxon x Treatment ($pCO_2$) | 1 | 779 | 2.27 | 0.1320 |

Type-I ANOVA of the linear mixed model testing the impact of Origin, Taxon and $pCO_2$ treatment on epidermal cell density (log-transformed).
