# Supplementary material for: Stomatal responses of differently CO2-acclimated plants to natural and experimental CO2 gradients
Source: PLoS One. 2026 Apr 22;21(4):e0346112. doi: 10.1371/journal.pone.0346112 (PMC13102186; doi:10.1371/journal.pone.0346112)
Supplement: S8 Table — Type-I ANOVA of the linear mixed model testing the impact of Origin, Taxon and pCO2 treatment on above-ground fresh weight. (PDF) [file pone.0346112.s010.pdf]

S8 Table. Response of above-ground fresh weight to variations in pCO_2.

**Above-ground fresh weight; n = 194**				
	numDF	denDF	F-value	p-value
Intercept	1	174	480.6730	<.0001
Origin	1	174	26.5751	<.0001
Taxon	1	174	81.4936	<.0001
Treatment (pCO_2)	1	6	30.4420	0.0015
Origin x Taxon	1	174	271.4817	<.0001
Origin × Treatment (pCO_2)	1	174	0.0701	0.7915
Taxon × Treatment (pCO_2)	1	174	1.1617	0.2826
Origin x Taxon x Treatment (pCO_2)	1	174	2.9551	0.0874

Type-I ANOVA of the linear mixed model testing the impact of Origin, Taxon and pCO_2 treatment on above-ground fresh weight.
