# Supplementary material for: Stomatal responses of differently CO2-acclimated plants to natural and experimental CO2 gradients
Source: PLoS One. 2026 Apr 22;21(4):e0346112. doi: 10.1371/journal.pone.0346112 (PMC13102186; doi:10.1371/journal.pone.0346112)
Supplement: Attachment — Submitted filename: Response to Reviewers 2.pdf [file pone.0346112.s013.pdf]

Response to Reviewers (PONE-D-25-38428)

We would like to thank the reviewer for the careful examination of our manuscript and the valuable suggestions for improvement. We are confident that these improvements will further enhance the quality of the article.

In the following, we will address each individual point raised by reviewer 2.

Author's replies to the comments of Reviewer 2

Reviewer 2: Comments to the Author

Reviewer #2: I found the revised version of the manuscript improved, well-written, and well-structured. I thank the authors for addressing my comments and concerns on the previous version. I have a few minor suggestions as below. Other than that, the manuscript is in a good shape.

Abstract
Should be one paragraph, not separated into multiple paragraphs.
Ok, changed

Introduction
L55: "to variations in Co2 levels" instead of "to Co2 levels variations".
Ok, changed

Materials and Methods
L205: "nutrient solution" instead of "nutrition solution".
Ok, changed

Statistical Analysis
Good.

Results
Good.

Tables
Good.

Discussion
Good.

Conclusions
Good.

Figures
I still think that statistical differences between treatments and/or species or lack thereof should be shown on the figures using labels or letters. I understand that overlapping and non-overlapping error bar tell the story, but it is always better to have the figures labeled appropriately so they are very straightforward and easy to understand by the reader without referring to the text. I think the Editor will agree with me on this.
To address the reviewer's concerns, we performed post hoc tests and marked the statistically significant differences in Figures 1, 3, 4, 5, and 6 with different letters. This procedure required some additional explanations, particularly in the “Methods” and “Results” sections. We hope that the figures are now clear and easy to understand.
