# Supplementary material for: Stomatal responses of differently CO2-acclimated plants to natural and experimental CO2 gradients
Source: PLoS One. 2026 Apr 22;21(4):e0346112. doi: 10.1371/journal.pone.0346112 (PMC13102186; doi:10.1371/journal.pone.0346112)
Supplement: Attachment — Submitted filename: The review report for the manuscript, Aug. 2025.docx [file pone.0346112.s011.docx]

The report on the review of the manuscript

**Stomatal responses of differently CO2-acclimated plants to natural and experimental CO2 gradients**

Adaptation of plants to environmental conditions, in particular to altitude ranges, is important for understanding the adaptation of photosynthesis processes. The results confirm the regulation of stomatal conduction in response to changes in the amount of CO2. It is possible to make a forecast of changes in the electrical conductivity of stomata with an increase in the level of CO2 in the atmosphere. The sample of the material is sufficient for interpretation and statistical analysis.

However, the question arises whether all plants will react equally to the CO2 content.

The tables are clear and understandable. The interpretation of the results was performed efficiently. The conclusions are logical. This follows from the results obtained and corresponds to the set goal.

References to literary sources are indicated correctly. The research is interesting and relevant. However, it is necessary to expand the list of plant objects in order to get a complete picture of the stomata's response to changes in CO2 levels.

I think the article should be published.

Reviewer: Doctor Oksana Belous
